# Supplementary material for: Preconditioning to Water Deficit Helps Aloe vera to Overcome Long-Term Drought during the Driest Season of Atacama Desert
Source: Plants (Basel). 2022 Jun 6;11(11):1523. doi: 10.3390/plants11111523 (PMC9183172; doi:10.3390/plants11111523)
Supplement: Supplementary file 1 [file plants-11-01523-s001.zip › Supplemental Table S1 and Figure S1.pdf]

Table S1. Monthly meteorological parameters in the study site during the period of preconditioning treatment (July-September) and subsequent water deprivation experiment (October-April). Data was obtained from a meteorological station located at the Canchones experimental field.

Parameter	Value	Month												
		Jan	Feb	Mar	Apr	May	Jun	Jul	Aug	Sep	Oct	Nov	Dec	Average
Temperature (°C)	Max	31.3	31.6	31.1	31.0	28.5	27.7	28.9	31.1	31.5	32.5	31.7	31.4	**30.7**
	Mean	21.5	19.9	19.5	17.7	14.4	12.5	12.9	16.0	17.4	18.5	18.3	19.7	**17.4**
	Min	12.8	8.7	9.2	6.4	2.1	-0.3	-1.0	2.4	4.7	5.6	5.5	8.6	**5.4**
Relative Humidity (%)	Max	68.9	66.6	70.9	69.6	70.0	66.5	50.9	47.1	60.9	59.1	68.8	65.7	**63.8**
	Mean	50.6	46.0	51.4	48.8	47.6	43.7	32.5	29.8	37.1	34.6	42.9	44.0	**42.4**
	Min	29.7	23.0	27.3	21.1	20.1	16.5	12.5	12.3	14.6	13.3	16.2	20.4	**18.9**
VPD (kPa)	Mean	1.29	1.27	1.11	1.06	0.88	0.84	1.04	1.31	1.27	1.42	1.23	1.30	**1.17**
Wind Speed (m s^{-1})	Max	4.7	4.5	4.3	3.1	2.2	1.8	1.8	2.9	3.7	4.1	4.2	4.6	**4.7**
	Mean	1.1	0.9	0.8	0.6	0.5	0.4	0.3	0.6	0.9	0.9	1.0	1.1	**0.7**
Precipitation (mm)	Mean	0.0	0.0	0.0	0.2	0.0	0.0	0.0	0.0	0.0	0.0	0.0	0.0	**0.2**
Solar Radiation (W m^{-2})	Max	1129	1150	1087	987	880	786	840	960	1091	1117	1158	1168	**1029.4**
	Mean	327.6	348.7	311.1	257.4	219.6	203.7	218.5	242.3	289.9	325.1	352.9	340.1	**286.4**
Irradiance (µmol photons $m^{-2}s^{-1}$)	Max	2508.89	2555.56	2415.56	2193.33	1955.56	1746.67	1866.67	2133.33	2424.44	2482.22	2573.33	2595.56	**2287.59**
	Mean	728	774.89	691.33	572	488	452.67	485.55	538.44	644.22	722.44	784.22	755.78	**636.46**
Reference ETo (mm/month)	Mean	272.1	261.0	254.0	209.2	173.4	151.2	170.8	210.0	244.3	282.1	285.8	288.4	**233.53**

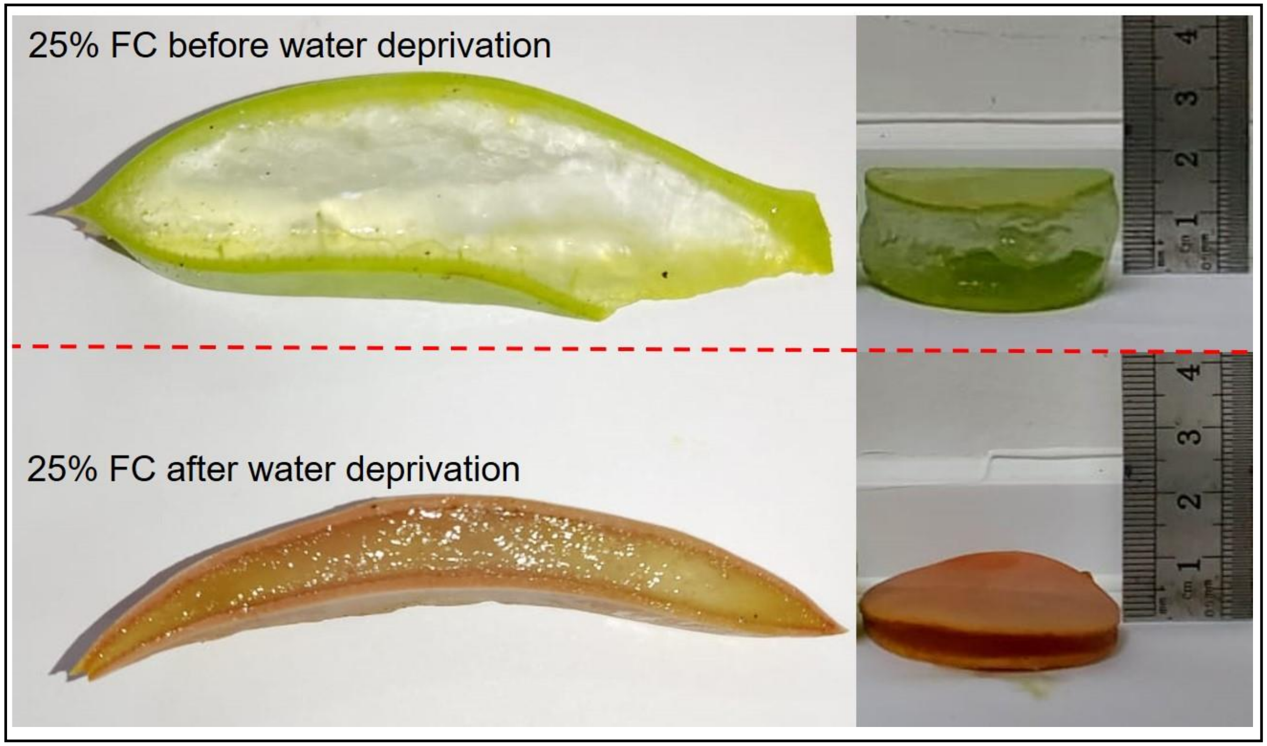

Figure S1. Transversal section of a leaf blade (left) and leaf discs of 10 cm^2 (right) from a plant preconditioned at 25% FC, before (above the red dashed line) and after (below the red dashed line) 222 days of water deprivation. Change in hydrenchyma thickness is evident, as well as the change from green to reddish color, likely due to the accumulation of “sunscreen” pigments such as anthocyanins and rhodoxanthin.
